# Supplementary material for: Amphiphilic PTB7-Based Rod-Coil Block Copolymer for Water-Processable Nanoparticles as an Active Layer for Sustainable Organic Photovoltaic: A Case Study
Source: Polymers (Basel). 2022 Apr 13;14(8):1588. doi: 10.3390/polym14081588 (PMC9029162; doi:10.3390/polym14081588)
Supplement: Supplementary file 1 [file polymers-14-01588-s001.zip › polymers-1663511-supplementary.pdf]

Supplementary Material

Amphiphilic PTB7-based rod-coil block copolymer for water-processable nanoparticles as active layer for sustainable organic photovoltaic: a case study

Marianna Diterlizzi[a,b], Anna Maria Ferretti[c], Guido Scavia[a], Roberto Sorrentino[a,†], Silvia Luzzati[a], Antonella Caterina Boccia[a], Andrea A. Scamporrino[d], Riccardo Po'[e], Eleonora Quadrivi[e], Stefania Zappia[a,*], Silvia Destri[a,*]

[a] *Istituto di Scienze e Tecnologie Chimiche "Giulio Natta" (SCITEC) - CNR, sede via A. Corti 12, 20133, Milano, Italy, marianna.diterlizzi@scitec.cnr.it; guido.scavia@scitec.cnr.it; r.sorrentino988@gmail.com; sil-via.luzzati@scitec.cnr.it; antonella.boccia@scitec.cnr.it;*

[b] *Dipartimento di Scienza dei Materiali, Università degli Studi di Milano-Bicocca, via Cozzi 55, I-20125 Milano, Italy,*

[c] *Istituto di Scienze e Tecnologie Chimiche "Giulio Natta" (SCITEC) - CNR, Laboratorio di nanotecnologie, sezione via G. Fantoli 16/15, 20138 Milano, Italy, anna.ferretti@scitec.cnr.it;*

[d] *Istituto per i Polimeri, Compositi e Biomateriali (IPCB) SS. Catania - CNR, Via P. Gaifami 18, 95126 Catania, Italy, andreaantonio.scamporrino@cnr.it;*

[e] *Eni SpA - Renewables, New Energies and Material Science Research Center, Istituto Eni Donegani, Via Fauser, 28100 Novara, Italy, riccardo.po@eni.com; eleonora.quadrivi@eni.com;*

[†] *Present address: STMicroelectronics S.r.l., Stradale Primosole 50, 95121 Catania, Italy*

**Correspondence: stefania.zappia@scitec.cnr.it; silvia.destri@scitec.cnr.it.*

Table of contents

Fourier transform infrared spectroscopy (FTIR) 3
Matrix assisted laser desorption/ionization time of flight mass spectrometry (MALDI-TOF MS) 4
2D-NMR Experiments 6
SEC analysis on the PTB7 macromer and the rod-coil BCP PTB7-b-P4VP 7
Differential scanning calorimetry (DSC) 9
Contact angle and energy surface measurements 10
GIXRD Experiments 11
WPNPs characterization 14
Device characterization 15

Fourier transform infrared spectroscopy (FTIR)

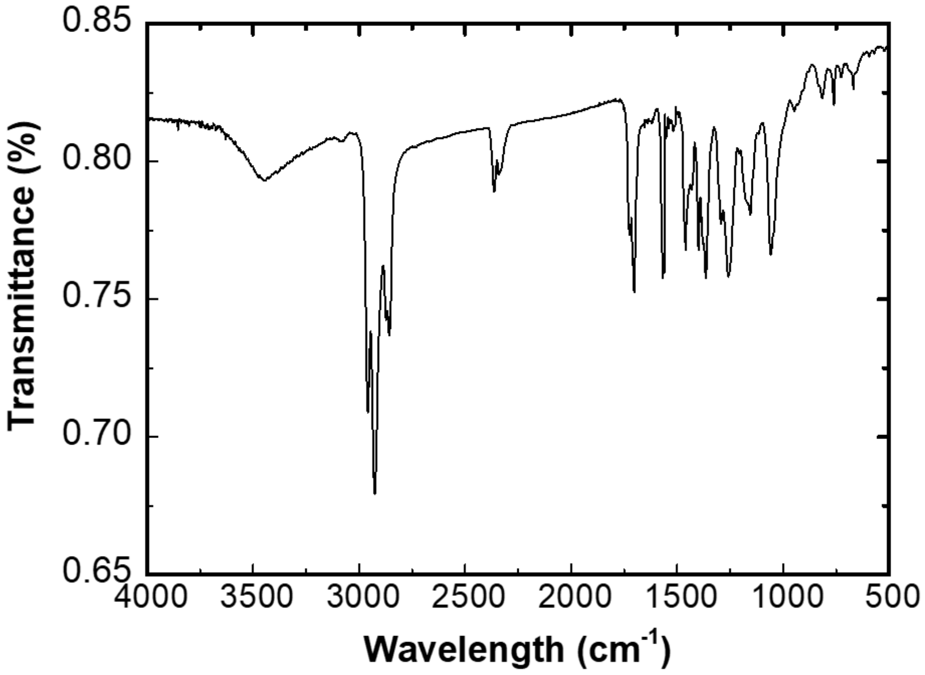

Figure S1 FTIR spectrum of the PTB7 macromer film on KBr disk.

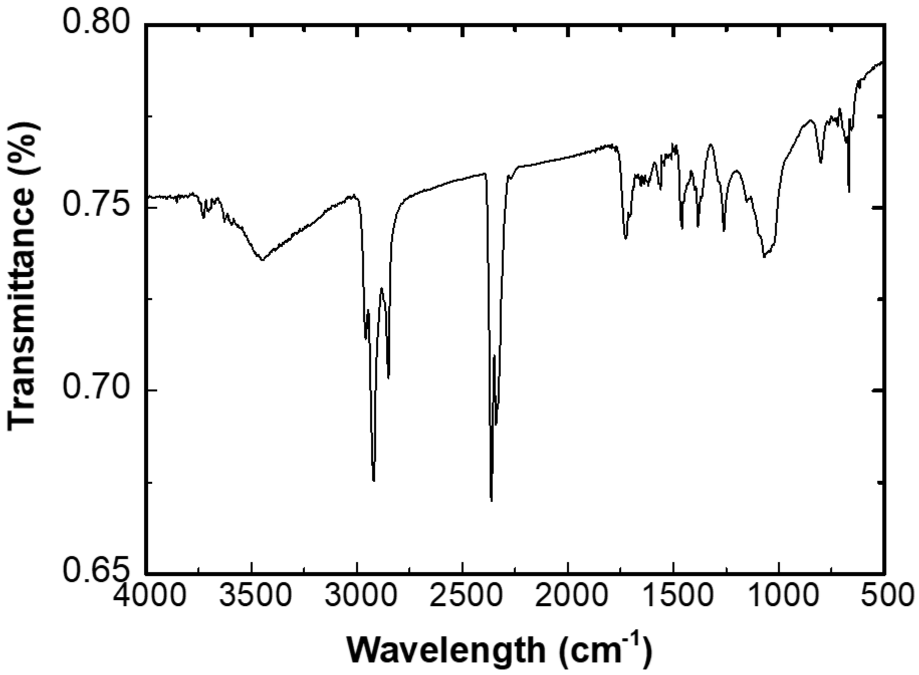

Figure S2 FTIR spectrum of the rod-coil PTB7-*b*-P4VP film on KBr disk.

Matrix assisted laser desorption/ionization time of flight mass spectrometry (MALDI-TOF MS)

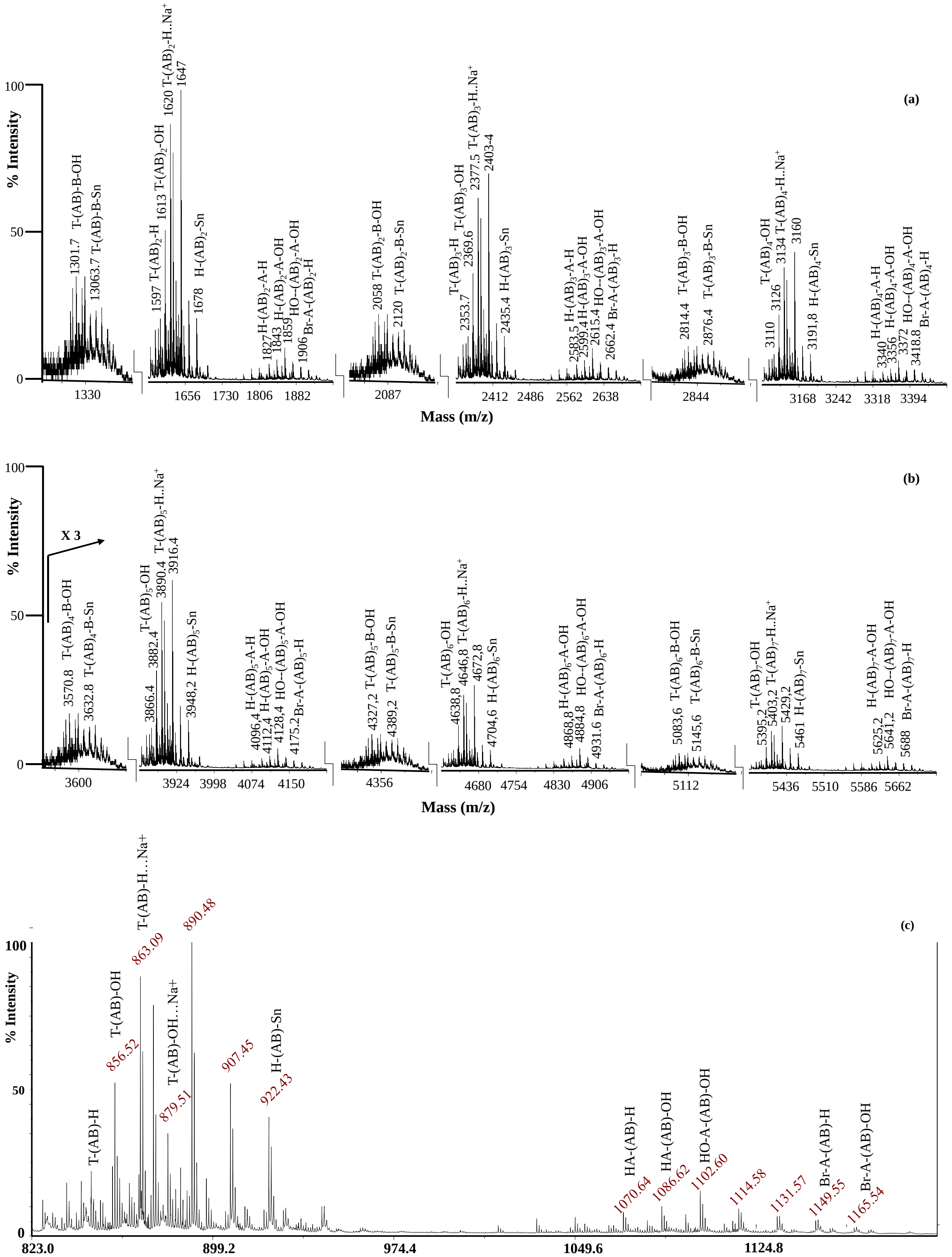

Figure S3. MALDI-TOF mass spectrum of PTB7 macromer in reflectron mode using DCTB as matrix ((a) and (b)), and its enlarged section (c).

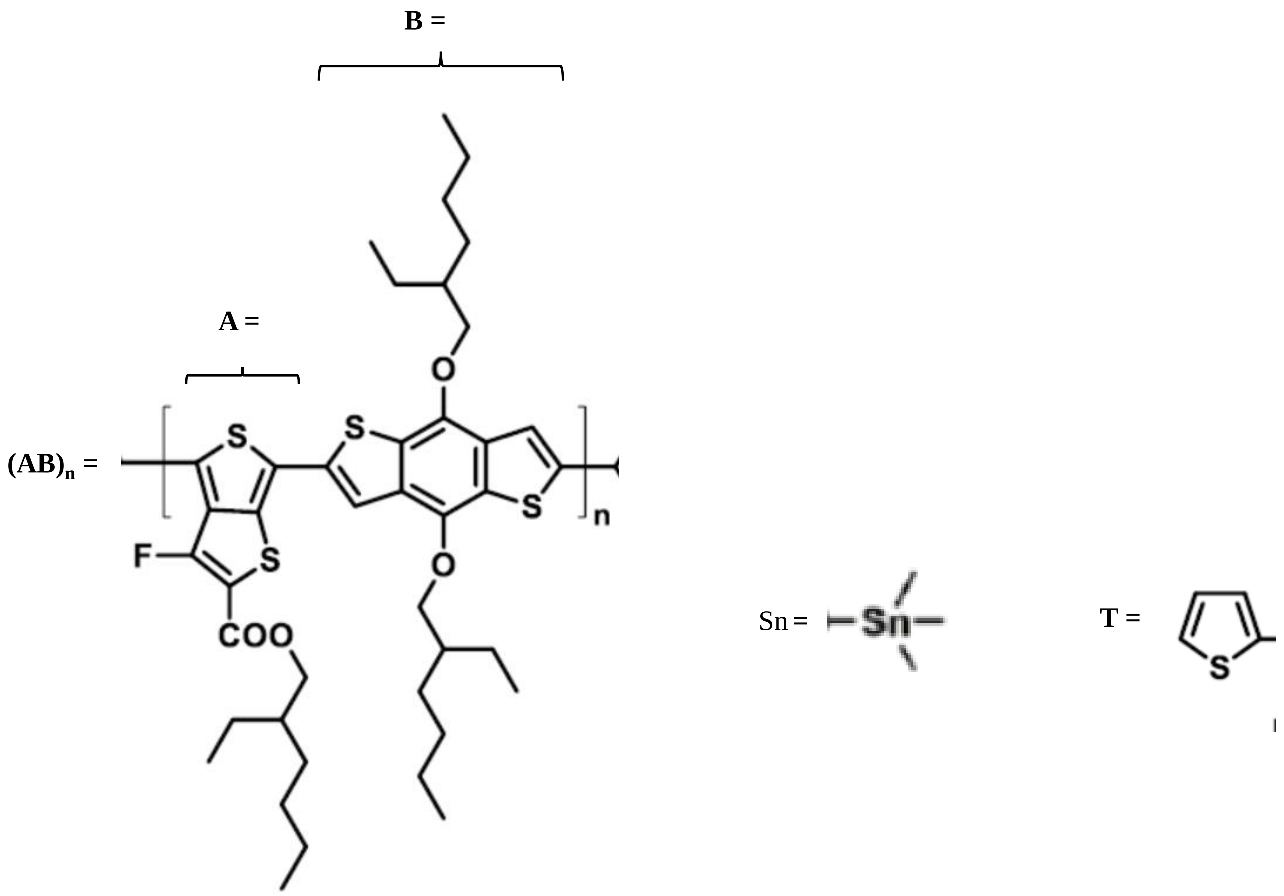

Chart S1 Structures of the PTB7 macromer chains and of their end groups as detected by MALDI-TOF MS analysis (**Figure S3**)

MALDI TOF mass spectrum of the PTB7 macromer (**Figure S3**) shows a series of repeating families of peaks spanning from m/z 820 up to ca. m/z 7800 showing 756.4 ± 0.1 Da transitions, corresponding to the mass of the repeating unit of the PTB7 homopolymer. The most intense peaks correspond to the molecular ions of the expected oligomers terminated with hydrogen, bromide and thiophene groups. Polymer chains terminated with hydroxyl and stannyl groups were also detected. The last one indicates the *living* behaviour of the PTB7 macromer. Moreover, the presence of the species H-A-(AB)-H, H-A-(AB)-OH, HO-A-(AB)-OH, Br-A-(AB)-H and Br-A-(AB)-OH shows that a direct arylation process occur during the synthesis of the PTB7 sample.

2D-NMR Experiments

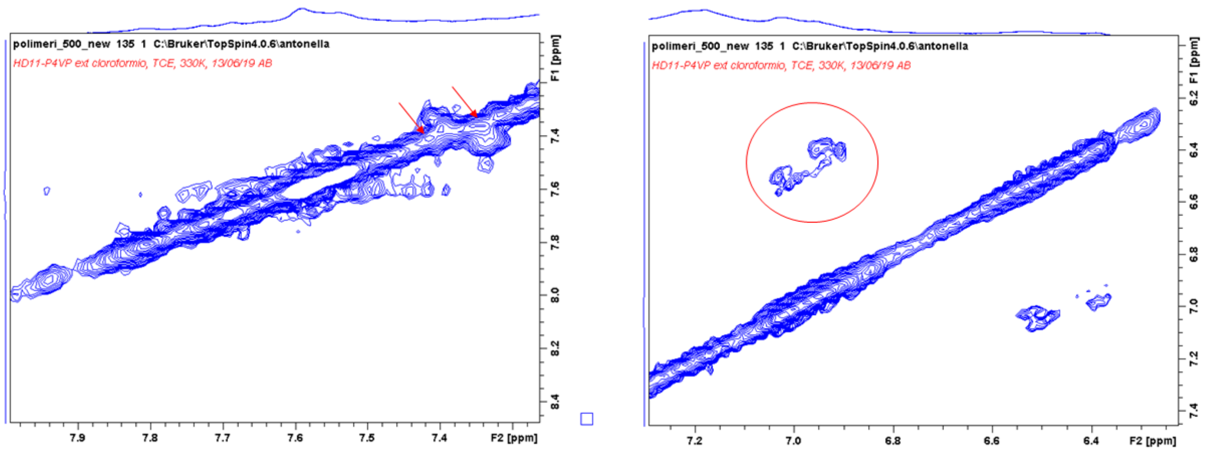

Figure S4. In this figure two section of the ^{1}H-^{1}H NMR TOCSY spectrum of the rod-coil BCP PTB7-*b*-P4VP are represented. In the left figure red narrows indicate signals that could be ascribable to correlations typical of phenyls. Besides, in the right section are circled signals which could be correlated to the aromatic protons adjacent to N atom of the P4VP segment. This signal is split because of the presence in the sample of chains with different length.

SEC analysis on the PTB7 macromer and the rod-coil BCP PTB7-b-P4VP

SEC analyses were carried out using the chromatographic system GPCV2000 Waters. GPCV2000 Water system is constituted by two different detectors: i) a differential refractometer (DRI) as concentration detector; ii) differential viscosimeter (DV). A Diode Array UV 996 was added to the SEC-DV system to register the UV-vis spectrum from 190 nm to 800 nm. Because of their different solubility, the samples PTB7 and PTB7-*b*-P4VP were characterized in a mixture THF:DMF=80:20 in order to compare the data obtained for the PTB7 macromer with the data obtained for the block copolymer. Experimental conditions: flow 0.8 mL/min; concentration ~2 mg/mL; injection volume: 220 μL. The other conditions were reported in the **Table S1**.

Table S1 SEC experimental condition related to the samples PTB7 and PTB7-*b*-P4VP.

	**P4VP**	**PTB7***	**PTB7***	**PTB7-*b*-P4VP**
**Mobile phase**	DMF+0.05M LiBr	80%THF+20% (DMF+0.05M LiBr);	THF	80%THF+20% (DMF+0.05M LiBr)
**Stationary phase**	2 Resipore PL	Resipore PL	3PL (Polipore, Oligopore, 50Å)	2 Resipore PL
**Temperature**	50 °C	35 °C	35 °C	35 °C

*PTB7 macromer

All the molecular weights were relative to a specific calibration. In particular, PTB7-*b*-P4VP and PTB7 macromer molecular weights are relative to polystyrene narrow standards and P4VP molecular weight is relative to PMMA narrow standards.

The MWD of the sample P4VP is very narrow. Furthermore, the molecular weight relative to PMMA suggests that the polymerization degree is very low (between 10 and 15 repeating units) in agreement with ^{1}H-NMR.

Table S2. MWD data of the coil P4VP (determined using DMF as the mobile phase) and of PTB7 macromer (determined using THF as the mobile phase) using DRI as detector.

**Sample**	**Mobile phase**	**M_n (g mol^{-1})**	**M_w (g mol^{-1})**	**M_w/M_n**
**P4VP**	DMF+0.05M LiBr	1100	1130	1.03
**PTB7 macromer**	THF	10337	25303	2.45

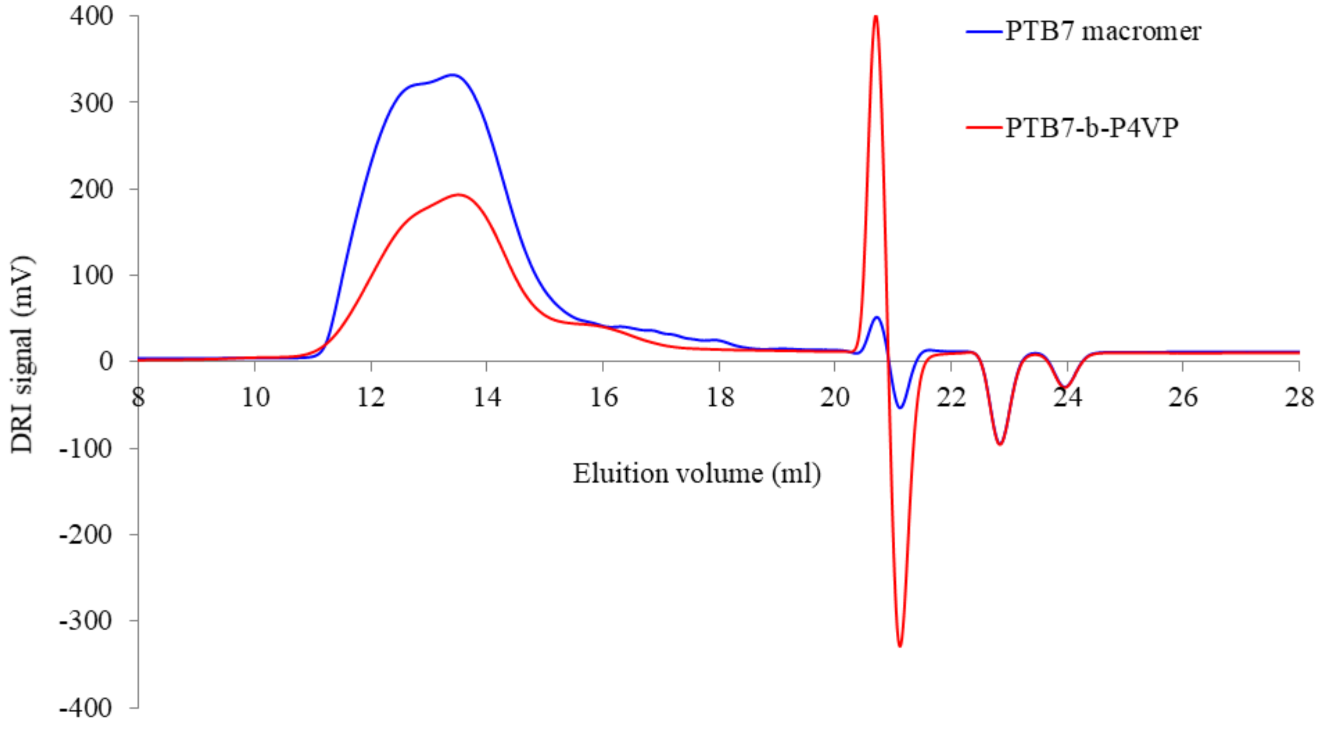

Figure S5 Chromatograms obtained by SEC analysis of the PTB7 macromer and the rod-coil PTB7-*b*-P4VP.

Differential scanning calorimetry (DSC)

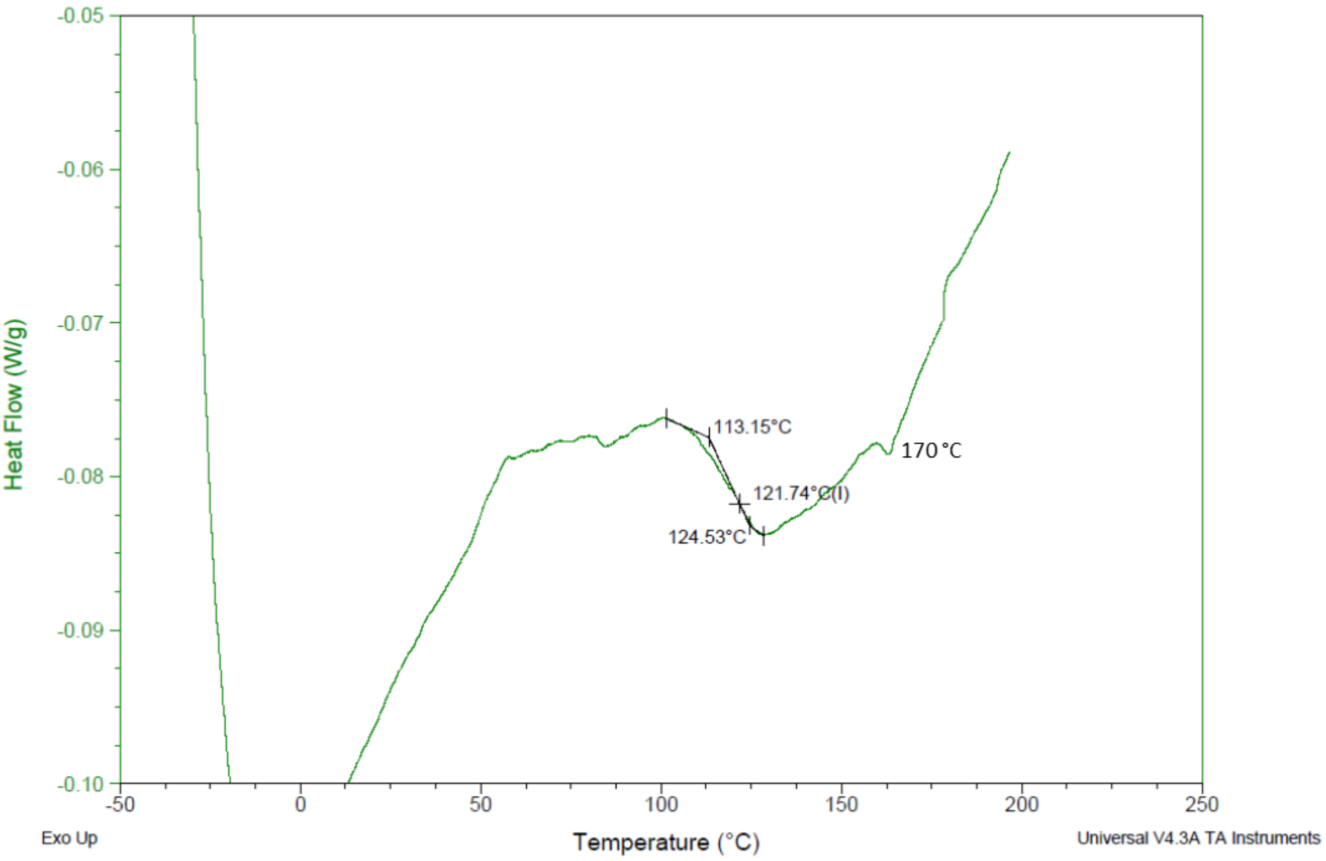

Figure S6 DSC thermogram of the PTB7-*b*-P4VP. Two peaks at ~120 °C and ~170 °C are distinguishable, related to the coil and the rod block, respectively.

Contact angle and energy surface measurements

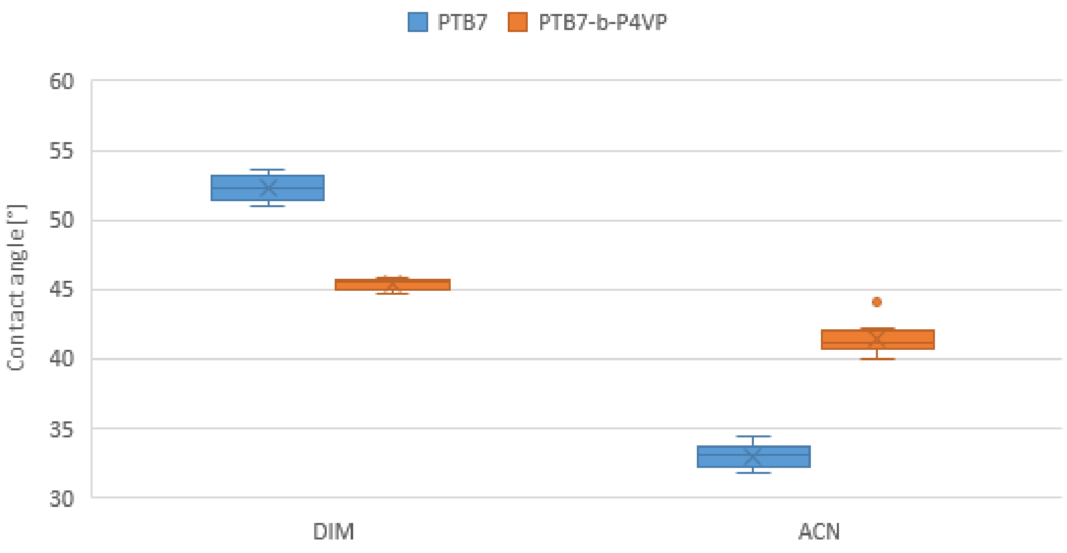

Figure S7 Distribution of contact angles of diiodomethane (DIM) and acetonitrile (ACN) on homemade PTB7 and PTB7-*b*-P4VP.

Table S3 Mean values and standard deviation of DIM and ACN contact angles on homemade PTB7 and PTB7-*b*-P4VP. For each solvent, mean and std. dev. are calculated from about 10 CA values

	**PTB7**	**PTB7-*b*-4VP**
**DIM [°]**	52.2 ± 0.9	45.4 ± 0.4
**ACN [°]**	33.0 ± 0.9	41.5 ± 1.2

GIXRD Experiments

GIXRD measurements were performed at the X-ray Diffraction beamline 5.2 at the Synchrotron Radiation Facility Elettra in Trieste (Italy). The X-ray beam emitted by the wiggler source on the Elettra 2 GeV electron storage ring was monochromatized by a Si(111) double crystal monochromator, focused on the sample and collimated by a double set of slits giving a spot size of 0.2 x 0.2 mm. The beam was monochromatized at 1.4 Å.

The samples were oriented by means of a four-circle diffractometer with a motorized goniometric head.

A diffractometer allowed sample surface alignment in the horizontal plane containing the X-ray beam by means of laser light reflection. For every sample, seven images at variable incidence (from -0.1° to 0.2°, step 0.05°) were taken, each one by rotating the sample of 360° around the normal to the surface in 60s of exposition to the beam.

The bidimensional diffraction patterns were recorded with a 2M Pilatus silicon pixel X-ray detector (DECTRIS Ltd., Baden, Switzerland) positioned perpendicular to the incident beam, 350 nm far from the sample.

Patterns were calibrated by means of a LaB6 standard and integrated using the software fit2d, obtaining powder-like patterns, corrected for geometry, Lorentz and beam polarization effects, in the azimuthal region between 70° and 110° for OOP signal and in that between 170° and 180° for IP signal.

Peaks positions were extracted by means of the program WinPlotr. Bidimensional images representing intensity as a function of q_{xy} and q_z, where q is the reciprocal lattice vector (or transferred momentum) expressed in $Å^{-1}$ were obtained with the software GIDVis.

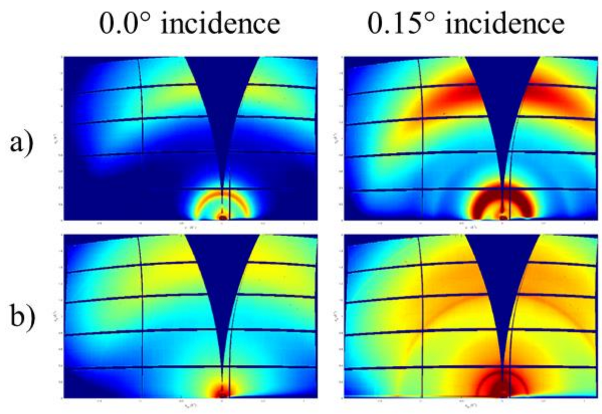

Figure S8 2D data from synchrotron radiation diffraction, at the incidence angle of 0.0° (right) and 0.15° (left) of a) a film obtained by drop-casting of homemade PTB7 (with molecular weight close to that used for BCP) dissolved into chloroform and b) PC71BM:PTB7-*b*-P4VP WPNP-based film.

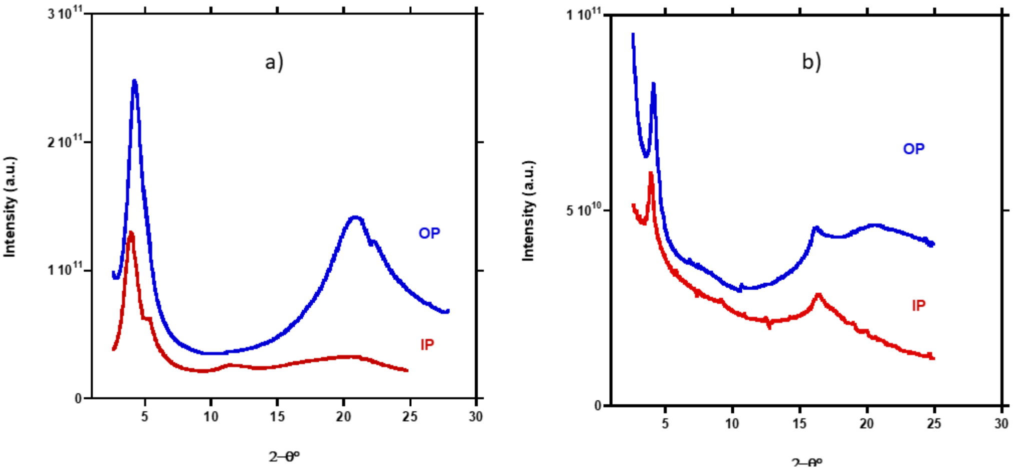

Figure S9 XRD profiles extracted from 2D images, as taken at an incident angle of 0.15° of a) a film obtained by drop-casting of homemade PTB7 (with molecular weight close to that used for BCP) dissolved into chloroform and b) $PC_{71}BM$:PTB7-*b*-P4VP WPNP-based film.

Table S4 GIXRD data of film **(a)** (obtained by drop-casting of homemade PTB7 with molecular weight close to that used for BCP dissolved into chloroform) and film **(b)** ($PC_{71}BM$:PTB7-*b*-P4VP WPNP-based film)

	**OOP peaks position (Å)**					**IP peaks position (Å)**			
Film (a)	18.93		3.85			20.35	7.40		3.89
Film (b)	19.37	4.86	3.86			20.21		4.90	

The comparison with the same polymer spin-coated reported by Hoefler et al. [1] indicates a more crystalline film, preferentially oriented "flat" onto the substrate, although significant amount of "edge-on" crystallites are evident as in OP profile interlayer spacing is observed, moreover the π-stacking shrinks from 0.4 (Hoefler polymer) to 0.39 nm (our films), although a significant contribution to this peak come from both glass substrate and amorphous part.

The comparison between 2D images taken at an incident angle close to 0.0° (S12 left) and at 0.15° (S12 right) reveals a larger order close to the substrate, as often observed for polymeric materials [2]. All the films are characterized by a long spacing at around 2 nm, while differences are observed at medium and short spacings, namely film (a) displays broad peaks centered at 0.7 nm and 0.39 nm (mainly in OP), while film (b) film shows peak at 0.49 nm together with 0.39 (in OP).

The 0.7 nm spacing in IP profile of film (a) could indicate a partial lateral stacking of crystallites edge-on oriented. In film (b), the usual peak attributed to $PC_{71}BM$ at 0.95 nm is hardly observable in OP profile indicating a quite disordered situation in the blend. The increase long spacing in film (b) is attributed to the presence of flexible P4VP. Noticeably the presence of relatively sharp peak at

0.486 nm, fourth order of long spacing, maps a significant degree of order attained especially along with the interlayer direction. On the other hand, the second order near to 0.95 nm (circa 10 Å) is completely overlapped by specific spacing of $PBC_{71}M$ broad in this case.

WPNPs characterization

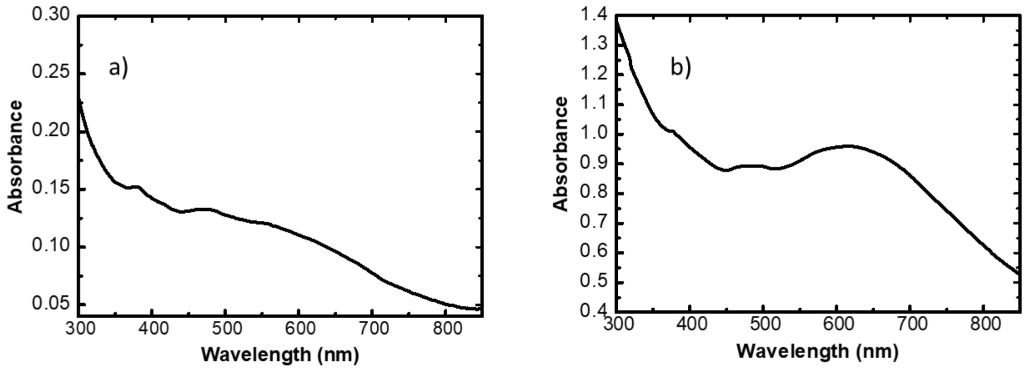

Figure S10 UV-Vis absorption spectra of $PC_{71}BM$:PTB7-*b*-P4VP (1.1:1) blend WPNPs obtained through a miniemulsion approach. Starting solution of the active materials obtained in **a)** toluene and in **b)** a mixture of toluene and *p*-xylene in ratio 50:50.

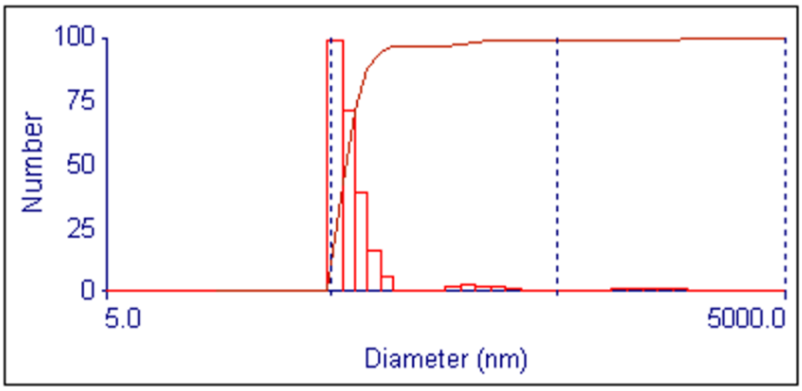

Figure S11 Multimodal size distribution by number of $PC_{71}BM$:PTB7-*b*-P4VP (1.1:1) WPNPs.

Device characterization

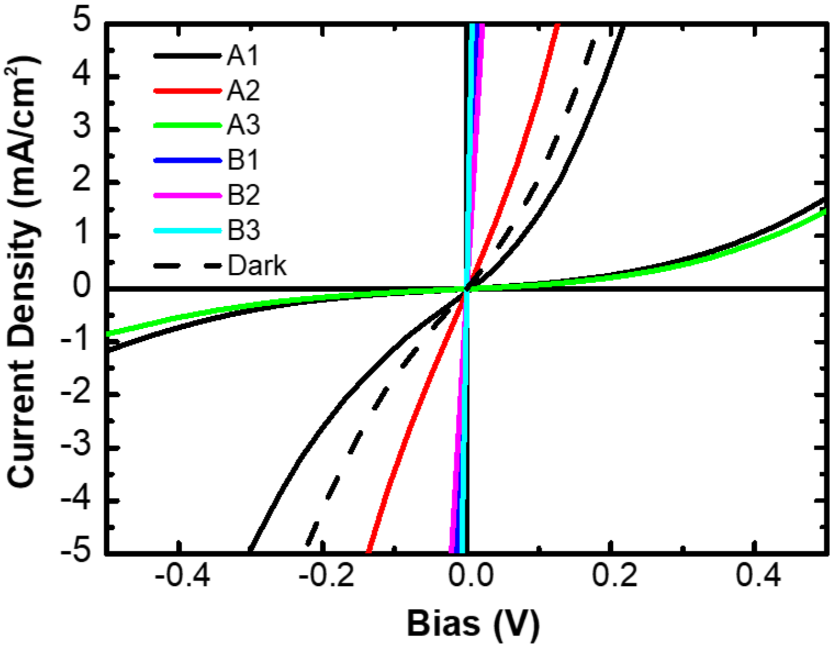

Figure S12 J-V curves of the device from $PC_{71}BM$:PTB7-*b*-P4VP (1.1:1) blend WPNPs with no interlayer on top.

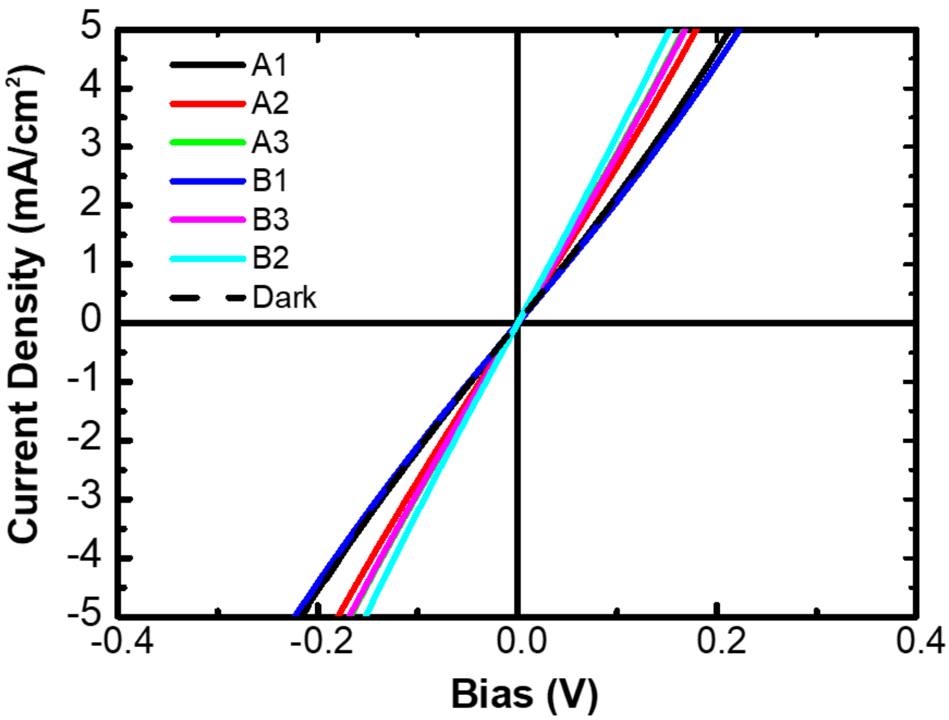

Figure S13 J-V curves of the device from $PC_{71}BM$:PTB7-*b*-P4VP (1.1:1) blend WPNPs with $PC_{61}BM$-PEG as interlayer (deposited from ethanol).

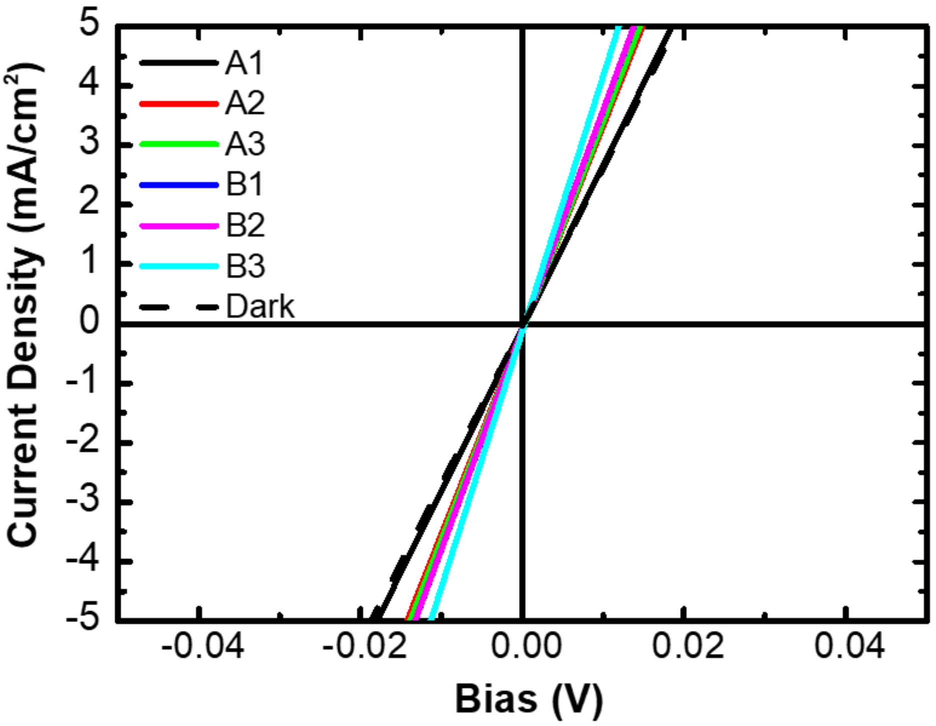

Figure S14 J-V curves of the device from $PC_{71}BM$:PTB7-*b*-P4VP (1.1:1) blend WPNPs with $PC_{61}BM$: $PC_{61}BM$-PEG (50:50) as interlayer (deposited from ethanol).

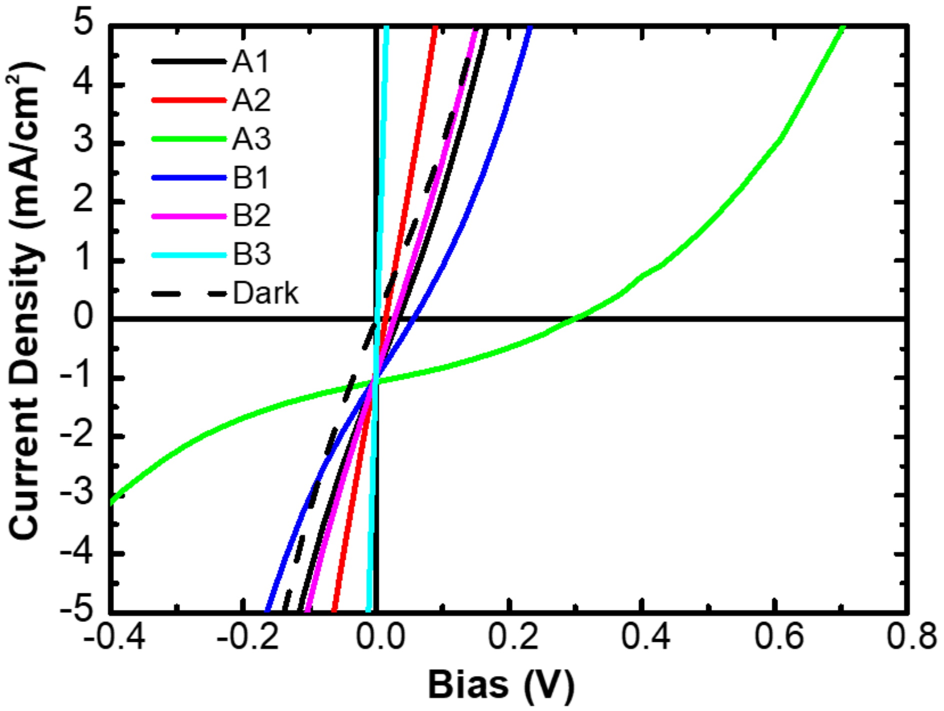

Figure S15 J-V curves of the device from $PC_{71}BM$:PTB7-*b*-P4VP (1.1:1) blend WPNPs with $PC_{71}BM$ as interlayer (deposited from dichloromethane).

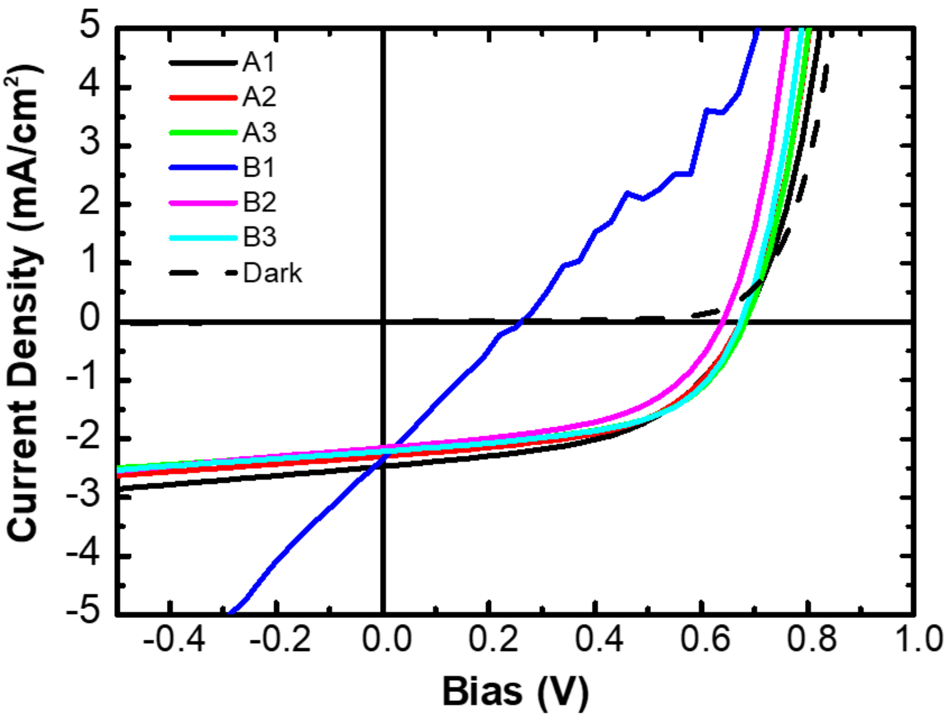

Figure S16 J-V curves of the device from $PC_{71}BM$:PTB7-*b*-P4VP (1.1:1) blend WPNPs with $PC_{61}BM$ as interlayer (deposited from dichloromethane).

References.

1. Hoefler, S.F.; Rath, T.; Pastukhova, N.; Pavlica, E.; Scheunemann, D.; Wilken, S.; Kunert, B.; Resel, R.; Hobisch, M.; Xiao, S.; et al. The effect of polymer molecular weight on the performance of PTB7-Th:O-IDTBR non-fullerene organic solar cells. *Journal of Materials Chemistry A* **2018**, *6*, 9506-9516, doi:10.1039/C8TA02467G.
2. Porzio, W.; Scavia, G.; Barba, L.; Arrighetti, G.; Milita, S. Depth-resolved molecular structure and orientation of polymer thin films by synchrotron X-ray diffraction. *European Polymer Journal* **2011**, *47*, 273-283, doi:https://doi.org/10.1016/j.eurpolymj.2010.12.007.
